# Efficacy and Safety of SBRT Combined With Camrelizumab and Apatinib in HCC Patients With PVTT: Study Protocol of a Randomized Controlled Trial

**DOI:** 10.3389/fonc.2020.01589

**Published:** 2020-08-26

**Authors:** Yue Hu, Tingting Qin, Shuang Li, Tao Zhang, Jun Xue

**Affiliations:** ^1^Cancer Center, Union Hospital, Tongji Medical College, Huazhong University of Science and Technology, Wuhan, China; ^2^Department of Epidemiology and Statistics, School of Public Health, Tongji Medical College, Huazhong University of Science and Technology, Wuhan, China; ^3^Department of Oncology, The First People’s Hospital of Jingzhou, Jingzhou, China

**Keywords:** HCC, PVTT, SBRT, camrelizumab, apatinib

## Abstract

**Background:**

Hepatocellular carcinoma (HCC) patients with portal vein tumor thrombosis (PVTT) has poor prognosis. Sorafenib/lenvatinib is recommended as the first-line therapy in these patients currently, with unsatisfactory response and survival benefit reported. Radiotherapy (RT) is increasingly utilized in advanced HCC and is considered an alternative option for HCC patients with PVTT. Combined treatment of RT and locoregional treatments such as transarterial chemoembolization shows promising results. However, the efficacy and safety for combined treatment of RT and systemic therapy have not been reported and thus warrant further studies. This prospective clinic trial aims at evaluating the efficacy and safety of stereotactic body RT (SBRT) combined with camrelizumab and apatinib in HCC patients with PVTT.

**Methods:**

This multicenter, open-label, randomized controlled trial will enroll 264 HCC patients with PVTT who have not received systemic therapy previously. Stratification of patients will be based on the presence or absence of extrahepatic metastasis and level of AFP (AFP ≥ 400 or <400 ng/mL) and randomly assigned 1:1 to study and control groups. Patients in study group will receive SBRT (95% PTV 36–40 Gy/6–8 Gy), camrelizumab (200 mg every 3 weeks), and apatinib (250 mg every day), and patients in control group will receive camrelizumab (200 mg every 2 weeks) and apatinib (250 mg every day). Patients will be followed up for 1.5 to 3.5 years since the start of therapy. We will use overall survival as the primary endpoint and progression-free survival, objective response rate, disease control rate, adverse events, and quality of life as the secondary endpoints.

**Discussion:**

This study will be the first randomized controlled trial to assess the efficacy and safety of SBRT combined with camrelizumab and apatinib for HCC patients with PVTT. The results may help establish a new standard first-line therapy for these patients.

**Trial Registration:**

Chinese Clinical Trial Registration No. ChiCTR1900027102.

**Date of Registration:**

October 31, 2019.

## Introduction

Liver cancer imposes a heavy disease burden and is the fourth leading cause of cancer-related mortality globally (1). Hepatocellular carcinoma (HCC) accounts for more than 80% of liver cancer (2). Almost 85% of HCCs occur in developing countries, especially in Eastern Asia and sub-Saharan Africa with am incidence rate of more than 20 per 100,000 individuals (2, 3).

Hepatocellular carcinoma is likely to invade the adjacent vasculature. Portal vein tumor thrombosis (PVTT) is the main form of macrovascular invasion with a prevalence rate ranging from 44 to 62% at autopsy (4). HCC patients with PVTT have an extremely poor prognosis, with overall survival (OS) as low as 2.7 to 4 months if untreated (5).

Currently, the treatment strategies for HCC patients with PVTT are still debated. The Barcelona Clinic Liver Cancer staging and management system considers HCC with PVTT at least advanced HCC (stage C). The standard care recommended for advanced HCC patients is systemic therapy, with the oral kinase inhibitors sorafenib or lenvatinib as the first-line treatment, and regorafenib or Nivolumab as the second-line therapy (5–7). In addition, multiple efforts have been invested in surgery, transarterial chemoembolization (TACE), radiotherapy (RT), and various combinations. The combined therapy strategies show promising results, however, we need prospective studies to validate the efficacy among HCCs with PVTT (4, 5).

Recently, the safety and efficacy of the combined treatment with programmed cell death 1 (PD-1)/PD-1 ligand (PD-L1) inhibitors plus molecular targeted medicine were demonstrated. A phase I clinic trial (NCT02942329) assessing the safety and efficacy of camrelizumab (anti–PD-1 monotherapy) combined with apatinib (VEGFR2 inhibitor) as a second line, or later treatment in advanced HCC showed that the objective response rate (ORR), disease control rate (DCR), and median time to response were 50.0% [95% confidence interval (CI) = 24.7–75.4%], 93.8% (95% CI = 69.8–99.8%), and 3.4 months (range, 1.4–9.7 months) (8). Besides, an earlier interim analysis of a phase Ib clinical trial (KEYNOTE 524 and NCT03006926) on the combined treatment of pembrolizumab plus lenvatinib in HCC patients showed that the ORR was 42.3% (95% CI = 23.4–63.1%), and the estimated median duration of progression-free survival (PFS) was 9.69 months (95% CI = 5.55 to not evaluable) (9, 10). The US Food and Drug Administration approved pembrolizumab plus lenvatinib as a potential first-line therapy for advanced unresectable HCC patients who were not eligible to locoregional therapy (9, 11).

Recent advances in RT technology had shown that external beam RT was an effective and safe alternative treatment for HCC patients, and its role was evolving from a palliative tool to a curative one. A randomized clinic trial compared the combined therapy of TACE and RT with sorafenib alone in untreated HCC patients with macroscopic vascular invasion. The results showed the TACE plus RT group had a significantly better prognosis than the sorafenib group, with median time to progression (31.0 vs. 11.7 weeks; *P* < 0.001) and OS (55.0 vs. 43.0 weeks; *P* = 0.04), respectively (12). Another randomized study compared neoadjuvant RT plus hepatectomy with hepatectomy alone in resectable HCC patients with PVTT. The results showed that the OS rates and the disease-free survival rates of the RT plus hepatectomy group were significantly higher than those of the hepatectomy alone group (13).

Although promising results had been demonstrated for the use of RT combined with locoregional treatments in HCC with PVTT, the efficacy and safety of RT with systemic therapy had not been reported. Thus, we attempt to perform a randomized controlled study to assess the efficacy and safety of stereotactic body RT (SBRT) combined with camrelizumab and apatinib as first-line therapy for HCC patients with PVTT.

## Methods and Analysis

### Study Design

This multicenter, open-label, randomized controlled trial will be conducted in 11 hospitals in China. The study has been authorized by the ethics committee of each center, and all the patients will provide written informed consent before registration. The trial has been registered in Chinese Clinical Trial Registry with number ChiCTR1900027102.

The PVTT diagnosis was made on typical radiological pattern identified on ultrasound, contrast-enhanced ultrasound, computed tomography (CT), magnetic resonance imaging (MRI), and/or histopathology findings (14). PVTT was classified into five groups based on Cheng’s classification: I0: microscopic tumor thrombus; I: tumor thrombus located in segmental or sectoral branches of the portal vein; II: right- or left-side branch of portal vein; III: main trunk of the portal vein; and IV: superior mesenteric vein (14, 15).

**FIGURE 1:**
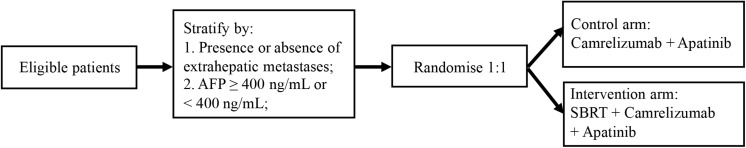
The study design of SBRT combined with camrelizumab and apatinib in HCC patients with PVTT.

Recruitment started in January 2020 and is estimated to continue until December 2021. Eligible HCC patients with PVTT are randomly allocated 1:1 to receive either camrelizumab, apatinib plus SBRT, or camrelizumab plus apatinib (Figure 1). Randomization will be performed using a minimization method with the following stratification factors: the presence or absence of extrahepatic metastases and level of AFP (AFP ≥ 400 ng/mL or <400 ng/mL). Monitoring will be carried out in this trial.

We will use OS as the primary endpoint and PFS, ORR, DCR, adverse events (AEs), and quality of life (QOL) as the secondary endpoints.

### Selection of Subjects

#### Eligibility Criteria

Patients with HCC diagnosed by histopathology or clinical criteria of European Association for the Study of Liver guidelines will be screened using the following inclusion and exclusion criteria (6). Noted that, in this study, tumor burden will not be considered; i.e., diffuse or multiple tumors invade both lobe of the liver, or huge tumor more than 10 cm in diameter; as long as they meet the following criteria, the patient could be enrolled.

#### Inclusion Criteria

• Patients willing to participate in the study and give written informed consent;
• patients aged ≥ 18 years;
• Cheng’s type II/III/IV PVTT;
• patients with recurrent HCC after locoregional treatment, such as hepatectomy, RT, TACE, hepatic artery perfusion, and radiofrequency ablation, which has been accomplished at least 4 weeks before the baseline imaging scan, and the grade of toxic reactions (except for hair loss) caused by the locoregional treatment should recover to less than 1 according to the National Cancer Institute–Common Terminology Criteria for Adverse Events (NCI-CTCAE) version 5.0;
• patients did not receive any systemic therapy previously;
• at least one HCC lesion which can be accurately measured on CT or MR images with at least one dimension ≥ 10 mm;
• ECOG performance status ≤ 1;
• Child–Pugh A;
• adequate hematological function: absolute neutrophil count ≥ 1.5 × 10^9^/L, hemoglobin ≥ 90 g/L, and platelet count ≥ 75 × 10^9^/L;
• adequate kidney function: creatinine < 1.5 × upper limit of normal (ULN), creatinine clearance rate > 50 mL/min;
• adequate hepatic function: albumin ≥ 29 g/L, total bilirubin ≤ 1.5 × ULN; alkaline phosphatase (AKP), aspartate transaminase, or alanine transaminase ≤ 5 × ULN; and
• patients with HBV infection will be enrolled if the HBV-DNA is less than 500 IU/mL or 2,500 copies/mL, and the patients receive at least 14 days of anti-HBV treatment before enrollment.

#### Exclusion Criteria

• Patients with cholangiocarcinoma, sarcomatoid HCC, mixed cell carcinoma, fibrolamellar cell carcinoma, or a history of other cancer in the past 5 years;
• patients who have moderate or severe ascites with clinical symptoms (i.e., those who need therapeutic puncture and drainage), or uncontrolled pleural effusion or pericardial effusion;
• patients with severe gastrointestinal bleeding, gastrointestinal perforation, or intestinal obstruction, or who were unable to swallow within 6 months before enrollment;
• patients with severe infection;
• patients with a history of embolism, cerebral infarction, or lung infarction;
• patients with a history of uncontrolled or unstable angina, uncontrolled hypertension, arrhythmias, cardiac insufficiency, congestive heart failure, or cardiac infarction occurring less than 6 months before registration;
• patients with interstitial pneumonia, interstitial lung disease, autoimmune diseases, innate, or acquired immune deficiency;
• systemic treatment with steroids or strong CYP3A4/CYP2C19 inducer or inhibitors within 14 days before enrollment;
• a history of serious drug allergy to monotherapy or targeted therapy;
• women who are pregnant or intend to become pregnant, or men whose partner is considering pregnancy; and
• patients who are currently enrolled in other investigational therapeutic drug or device studies.

### Interventional Methods

#### Immunotherapy

Each cycle of the treatment will be 6 weeks. A fixed dose of 200 mg camrelizumab will be administered intravenously every 3 weeks. In the study group, the first dose of camrelizumab will be given within 7 days after; in the control group, subjects will receive camrelizumab on day 1 of cycle 1. Camrelizumab administration will continue until intolerable toxicity or disease progression occurs. If AE, laboratory test abnormality or intercurrent disease happens, camrelizumab treatment will be delayed, but the dosage cannot be reduced. Treatment interruption will be allowed for no more than 12 weeks (either continuously or intermittingly); otherwise, the patients will be withdrawn from the study. Patients with progressive disease will continue the treatment if the investigator estimates they may still get clinical benefit and will be reevaluated after each cycle (6 weeks).

#### Targeted Therapy

The dose of oral apatinib will be 250 mg once daily. Similarly, the first dose of apatinib will be given within 7 days after SBRT for the patients of study arm and will be given on day 1 of the first cycle for the patients of control arm. Dosage modifications (first dose reduction: 200 mg, 5 days on, 2 days off; second dose reduction: 200 mg, every 2 days) or treatment suspension will be allowed, resulting from grade 2 non-hematologic, or grade 3 hematologic toxicity. Dosage modifications could be made twice in every cycle (6 weeks). However, once the dose reduces, it could not be re-escalated.

Apatinib will be discontinued when the toxicity is still intolerable through two dose modifications.

For both camrelizumab and apatinib, treatment cycles will be continued until unacceptable toxicity or disease progression occurs or patient’s request for withdrawal from the study. After discontinuation of apatinib, subjects will be allowed to continue to receive camrelizumab, and *vice versa*.

#### Radiotherapy

Stereotactic body RT will be started within 1 week after enrollment for the patients of study group. 4DCT will be used for treatment planning and evaluating the tumor motion. The gross tumor volume (GTV), defined by contrast-enhanced CT or MRI, will encompass the tumor and PVTT if this can meet the dose-volume constraints for the organs at risk. Otherwise, only PVTT will be regarded as the GTV (16). The clinical tumor volume (CTV) is produced by expanding GTV with 5 to 10 mm. The planning target volume (PTV) is constructed by adding 5 to 10 mm to CTV in all directions. A total of 36 to 40 Gy for the PTV with the fraction size of 6 to 8 Gy will be administered by using 6-MV x-rays with a linear accelerator at five fractions per week (Varian Medical Systems).

The radiation dose volume constraints for organs at risk are as follows: for liver, total spared volume (*V*_total_-*V*_15 Gy_) should be more than 700 mL and/or *V*_15 Gy_ should be less than 1/3 *V*_total_; for spinal cord, *V*_1 mL_ should be less than 15 Gy; for stomach and small bowel, *V*_1 mL_ should be less than 5 Gy; for duodenum, *V*_1 mL_ should be less than 25 Gy; for kidneys, 1/3 *V*_total_ should be less than 15 Gy (17).

### Assessment

#### Tumor Response Assessment

Baseline radiological scan will be performed within 28 days prior to the first study treatment. During the treatment period, imaging assessment for efficacy will be conducted every 6 weeks. Baseline and each subsequent assessment must follow the same radiological procedures, which include chest CT and abdomen and pelvis MRI. Brain MRI is also required at baseline, but is not necessary during subsequent tumor assessment if tumor was not detected initially. RECIST v1.1 is utilized for assessment of treatment response. At the discretion of investigators, radiological scans should be repeated at any time if progression is suspected. Patients who discontinue study treatment without progression (e.g., AEs) will be followed for tumor assessments until the patients experience progression, withdraw consent, die, or until the study terminates, whichever occurs first. Patients who continue camrelizumab/apatinib treatment beyond radiographic disease progression will be monitored with a follow-up scan at least 6 weeks later or at the next scheduled tumor assessment. For patients who continue treatment based on investigator assessment of clinical benefit, tumor assessment will continue until treatment discontinuation.

#### Quality of Life

Two questionnaire [the European Organization for Research and Treatment of Cancer Quality of Life Questionnaire C30 (EORTC-QLQ-C30) and the European Organization for Research and Treatment of Cancer Hepatocellular Carcinoma Quality of Life Questionnaire 18 (EORTC-QLQ-HCC18)] will be utilized to assess the QOL scores at baseline and each follow-up (18).

### Follow-Up

A safety follow-up visit is required for each patient after treatment discontinuation. Patients who show progression will have the safety follow-up at the last follow-up visit when the response assessment shows progression and results in treatment discontinuation. Patients who discontinue treatment for any reason need to return to the clinic for a safety visit within 30 ± 7 days since their last treatment in the case of a treatment-emergent AE.

After study treatment discontinuation, every patient will be followed up for study drug–related serious AEs (SAEs) and survival status. These follow-ups will start 3 months after the safety visit and be done every 3 months until loss to follow-up, death, consent withdrawal, or trial termination by the investigator. Information of survival status and subsequent antitumor treatment will be collected via telephone calls, patient medical records, and/or clinic visits.

### Sample Size

Sample size of the study is calculated using log-rank test. The estimated OS is 11 months for the study arm and 7 months for the control arm (8, 19–21). The estimated study duration will be 3.5 years, including 2 years of recruitment, and 1.5 years of follow-up. The upper limit of 95% CI of hazard ratio is 0.636 (two-sided) with 5% probability errors and 80% power. Thus, the total sample size required is 264 (132 per arm) after taking into account of a 5% dropout rate.

### Statistical Analysis

Statistical analyses will be performed with SAS software (version 9.3; SAS Institute, Cary, NC, United States). *P* < 0.05 will be interpreted as significant, and we will be able to reject the null hypothesis. Kaplan–Meier curves will be plotted to compare OS and PFS between the two groups by means of log-rank test. Duration of overall response (DOR) and ORR will be calculated based on binomial distributions using two-sample Cochran–Mantel–Haenszel method. Independent-sample *t* test or Wilcoxon rank-sum test will be applied to compare QOL score between both groups. Descriptive statistics will be analyzed for safety data.

### Outcome Definitions

• Overall survival: duration from randomization to death (regardless the cause);
• Progression-free survival: duration from randomization to progression or death whichever is earlier;

• Objective response rate: percentage of patients with a complete response (CR) or partial response (PR; using RECIST 1.1) and for a minimum duration, usually measured from the of treatment initiation to disease progression;
• Disease control rate: the proportion of patients who achieve CR, PR, or stable disease (SD);
• Adverse events: AEs will be record based on NCI-CTCAE Version 5.0;
• Quality of life: QOL scores will be measured according to the QLQ-C30 and QLQ-HCC 18 questionnaire.

## Anticipated Results

Overall survival will be used as a primary endpoint, and PFS, ORR, DCR, AEs, and QOL will be as secondary endpoints. We expect that the OS, PFS, ORR, and DCR of the study group will be significantly better than those of the control group, but there will be no difference for AEs and QOL.

## Discussion

Portal vein tumor thrombosis is one of the most ominous prognostic factors in HCC. Not only may tumor thrombus cause intrahepatic tumor dissemination, but it can also rapidly decrease blood flow to the liver, resulting in portal hypertension and deterioration of liver function reserve. This, in turn, may reduce tolerance to treatment (4, 14). Although varied therapeutic patterns have been recommended for HCC patients with PVTT, the optimal treatment is still undetermined.

Because HCC tumor thrombus progresses very quickly, fast lessen tumor thrombus volume will be helpful for the following treatment of the primary cancer lesion. It has demonstrated that ORR is changing from 39 to 62% for RT in HCC patients with PVTT (12, 22–24). Although RT alone could achieve a high locoregional tumor control rate, combining systemic treatments with RT seems necessary because of the failure outside the radiation field (12, 25). Proper tumor oxygenation is good for enhancing the RT efficacy, so improvement of tumor hypoxia has been explored using antiangiogenic agents. Besides, combined immunotherapy with RT could have independent antitumor efficacy and may induce the radiation abscopal effect. A well-known mechanism for the enhancement of antitumor immune response is that RT could recruit immune cells and induce the death of immunogenic cell (26). Previous studies showed us a synergistic effect between antiangiogenic agents and immunotherapy (camrelizumab plus apatinib or pembrolizumab plus lenvatinib) (8, 9). PD-1/PD-L1 blockade could sensitize tumors to antiangiogenic treatment and increase its efficacy, and the latter could also facilitate PD-1/PD-L1 therapy because induction of intratumoral high endothelial venules will enhance infiltration and activity of cytotoxic T lymphocytes (26).

However, prospective studies investigating these observations are scarce. In this clinic trial, we sought to evaluate the efficacy and safety of combination SBRT with camrelizumab and apatinib in the HCC patients with PVTT. If the anticipated results above could be achieved, it would provide strong evidence of a new first-line therapy in HCC patients with PVTT.

## Data Availability Statement

The original contributions presented in the study are included in the article/supplementary material, further inquiries can be directed to the corresponding authors.

## Ethics Statement

The studies involving human participants were reviewed and approved by China Ethics Committee of Registering Clinical Trials. The patients/participants provided their written informed consent to participate in this study.

## Author Contributions

JX and TZ devised the study concept and design. YH drafted the manuscript. TQ was responsible for the statistics. SL, TQ, and YH have made substantial contribution to the study protocol. JX, TZ, SL, TQ, and YH reviewed and approved the final version of this article. All authors contributed to the article and approved the submitted version.

## Conflict of Interest

The authors declare that the research was conducted in the absence of any commercial or financial relationships that could be construed as a potential conflict of interest.

## Abbreviations

95% CI: 95% confidence interval
AEs: Adverse events
CTV: Clinical tumor volume
DCR: Disease control rate
HCC: Hepatocellular carcinoma
MRI: Magnetic resonance imaging
NCI-CTCAE: National Cancer Institute–Common Terminology Criteria for Adverse Events
ORR: Objective response rate
OS: Overall survival
PFS: Progression-free survival
PVTT: Portal vein tumor thrombosis
QOL: Quality of life
RT: Radiotherapy
SBRT: Stereotactic body radiotherapy
TACE: Transarterial chemoembolization
ULN: Upper limits of normal.
